# Handling Data Skew in MapReduce Cluster by Using Partition Tuning

**DOI:** 10.1155/2017/1425102

**Published:** 2017-03-29

**Authors:** Yufei Gao, Yanjie Zhou, Bing Zhou, Lei Shi, Jiacai Zhang

**Affiliations:** ^1^College of Information Science and Technology, Beijing Normal University, Beijing, China; ^2^Department of Industrial Engineering, Pusan National University, Pusan, Republic of Korea; ^3^Cooperative Innovation Center of Internet Healthcare, Henan Province, China; ^4^School of Information Engineering, Zhengzhou University, Zhengzhou, China; ^5^Beijing Advanced Innovation Center for Future Education, Beijing Normal University, Beijing, China

## Abstract

The healthcare industry has generated large amounts of data, and analyzing these has emerged as an important problem in recent years. The MapReduce programming model has been successfully used for big data analytics. However, data skew invariably occurs in big data analytics and seriously affects efficiency. To overcome the data skew problem in MapReduce, we have in the past proposed a data processing algorithm called Partition Tuning-based Skew Handling (PTSH). In comparison with the one-stage partitioning strategy used in the traditional MapReduce model, PTSH uses a two-stage strategy and the partition tuning method to disperse key-value pairs in virtual partitions and recombines each partition in case of data skew. The robustness and efficiency of the proposed algorithm were tested on a wide variety of simulated datasets and real healthcare datasets. The results showed that PTSH algorithm can handle data skew in MapReduce efficiently and improve the performance of MapReduce jobs in comparison with the native Hadoop, Closer, and locality-aware and fairness-aware key partitioning (LEEN). We also found that the time needed for rule extraction can be reduced significantly by adopting the PTSH algorithm, since it is more suitable for association rule mining (ARM) on healthcare data.

## 1. Introduction

Healthcare is a highly data-intensive industry where data are driven by record keeping, compliance and regulatory requirements, and patient care [1]. These diverse data include radiology images, clinical records, human genetics records, and population data genomic sequences. The use of big data analytics in healthcare offers many attractive opportunities while posing significant challenge. However, traditional data processing and analytical algorithms cannot satisfy the requirements of big healthcare data and cloud computing. Fortunately, advances in data management, particularly such parallel computational models as MapReduce, can be applied to process and analyse diverse and large-scale datasets. However, big data are so large and complex that they cannot be managed under traditional methods. For example, when using association rule mining (ARM) on MapReduce, algorithms must extract the necessary information from big data in a timely manner. MapReduce is a powerful and cost-effective tool for massively parallel analytics. It can distribute data and computational tasks to thousands of cheap physical nodes, hence providing massive storage capacity and parallel computing capabilities [2]. MapReduce is a programming model that allows the easy development of scalable parallel applications to process big data on large clusters of commodity machines [3]. A MapReduce job typically runs in two main phases: a map phase and a reduce phase. In each phase, distributed tasks process datasets on a cluster of computers. When a map task is completed, the reduce tasks are notified to pull newly available data. This transfer process is referred to as a shuffle. All map tasks must be completed before the shuffle part of the reduce phase to allow the latter to complete. We consider a case where computational load is unbalanced among map tasks or reduce tasks. We call such an unbalanced situation map skew or reduce skew, respectively. Skew can lead to longer job execution times and lower cluster throughput, thus affecting the performance of MapReduce. Kwon et al. [4] analysed the types of skew that arises in a variety of MapReduce applications but did not provide a relevant solution to unbalanced partitioning in the reduce phase. Ibrahim et al. designed the LEEN algorithm [5] to determine the corresponding partition of a map output based on the frequency of key-value pairs. When a large amount of data and keys are unevenly distributed, data skew may occur, resulting in an unbalanced input of reduce tasks. Xu et al. [6] focused on presampling partitioning strategy to deal with unbalanced partitioning in the reduce phase. However, when dealing with massive amounts of data, the sampling overhead incurred by this strategy is high and affects the performance of MapReduce. Ramakrishnan et al. [7] proposed techniques to split each key with a large record size into subkeys to allow for a more even distribution of workload among reducers. However, it requires waiting until all map tasks are completed to gather partition size information before reduce tasks can begin.

Lin [8] found that using the default hash partitioning method, nearly 92% of reduce tasks yielded data skew, and the running time of reducers was 22% to 38% higher in general than normal tasks. Hence, we focus on reducing skew and trying to improve the partitioning method in the shuffle phase. Gufler et al. [9] proposed a one-stage partitioning method called “Closer,” which depends on the sampling information of the distribution. It divides the skew partitions and recombines them to attain a balance. The difficulty of this method is in dividing the skewed partitions properly, and the amount of time needed can seriously affect performance. The one-stage partitioning strategy involves the use of only the hash/range function to divide tuples in the map phase and randomly assigns partitions to the corresponding reducer. This strategy can achieve balance in each reducer, but for skewed data, the default partition method finds it difficult to balance the data for one-time uniform division. Considering this issue, we want to use a two-stage strategy to divide the map output into fine-grained partitions and recombine them based on global output information to disperse skewed data. In this paper, we propose a data processing algorithm called Partition Tuning-based Skew Handling (PTSH) to address the problem. First, we first use a virtual partitioning method to divide the original partitions into fine-grained partitions and collect real-time stats regarding the data size of each partition. Second, the partitioning information of the map task is extracted and the corresponding index sent to the reduce tasks for repartition. Finally, the repartitioning process divides the collected virtual partitions into new partitions of the same number as the reduce tasks. The main contributions of the paper lie on the following:

1. Based on a two-stage partitioning strategy, we propose a partition tuning method to divide skewed partitions into fine-grained partitions and use a repartition method to solve the problem of unbalanced data division. As partitioning is an NP-hard problem, we propose a repartition algorithm, which can effectively balance skewed partitions.
2. We conducted several experiments on simulated datasets and real datasets. Compared with one-stage strategies, the results showed that our method could effectively mitigate data skew in MapReduce jobs and improve efficiency.
3. A case study of ARM for real healthcare data was carried out on MapReduce. Combining an Apriori algorithm and PTSH, it could balance the data distribution of reduce tasks and improve the efficiency of ARM on healthcare data.

The rest of this paper is organized as follows: We report the background for this study in Section 2 and present the proposed PTSH approach in Section 3. The performance evaluation of PTSH and the case study are reported in Section 4, and we draw our conclusions and provide suggestions for future work in Section 5.

## 2. Background

In this section, we provide the background for the MapReduce programming model, data skew in ARM on MapReduce, and the partition skew problem therein that motivates our study.

### 2.1. MapReduce Programing Model

MapReduce automatically parallelizes and executes a program on a large cluster of commodity machines. It works by breaking processing into two phases, the map phase and the reduce phase. Each phase has key-value pairs as input and output, the types of which may be chosen by the programmer. The map and reduce functions of MapReduce are both defined with respect to data structured in (key, value) pairs. The computation takes a set of input key-value pairs and produces a set of output key-value pairs. The map and reduce functions in Hadoop [10] MapReduce have the following general form:

(1) $$\begin{array}{c} \begin{array}{c} map\text{: }\left(k_{1},v_{1}\right)\to list\left(k_{2},v_{2}\right)\\ \text{reduce: }\left(k_{2},list\left(v_{2}\right)\right)\to list\left(v_{2}\right). \end{array} \end{array}$$

**Figure 1:**
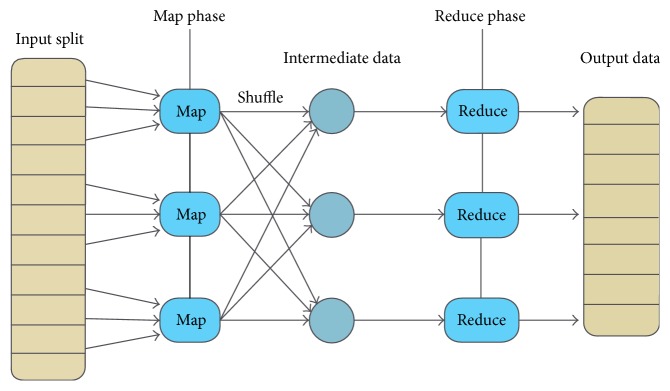
MapReduce programing model.

When a MapReduce job starts, the map invocations are distributed across multiple machines through the automatic partitioning of input data into a set of splits. As shown in Figure 1, the map tasks take input splits as inputs and generate a sequence of key-value pairs called intermediate data. A partitioning function (by default, hash(key) mod R) is then used to divide the intermediate data into several partitions and distribute them across reduce tasks. This transfer process is called a shuffle. In the reduce phase, each reduce task processes the input intermediate data by the reduce function and generates output data.

In this study, we focus on the shuffle process in the MapReduce programing model because data skew invariably occurs in this period and seriously affects the performance of MapReduce.

### 2.2. Data Skew in ARM on MapReduce

Data mining is the computational process of discovering patterns in large datasets involving methods at the intersection of artificial intelligence, machine learning, statistics, and database systems. The overall goal of the data mining process is to extract information from a dataset and transform it into an understandable structure for further use. Data mining nowadays has become popular in healthcare because of the need for an efficient analytical methodology to detect unknown and valuable information in healthcare data [11]. Association is one of the most vital approaches to data mining used to determine frequent patterns and other interesting relationships among a set of data items in a repository. Association has a significant impact on healthcare in detecting relationships among diseases, patient statuses, and symptoms. Ji et al. used association to discover infrequent causal relationships in electronic healthcare databases [12]. Patil et al. [13] used an Apriori algorithm to generate association rules to classify patients suffering from type 2 diabetes. Abdullah et al. [14] proposed a modification in an existing Apriori algorithm to add information to medical bills.

Efficiency is the most important factor in association mining. Parallel algorithms for ARM are not suitable for high-dimensional and large amounts of data because they are susceptible to data placement problems, which lead to skew [15]. For MapReduce, data skew is an important problem adversely affecting load balancing in ARM algorithms. It partitions the dataset horizontally in blocks of equal size. However, the number of frequent itemsets generated from each block can be heavily skewed, that is, while one block may contribute many frequent itemsets, another may have very few, implying that the processor responsible for the latter block is idle most of the time. Another kind of data skew occurs if itemsets are frequent in many blocks, or if they are frequent in only a few blocks. Hence, the algorithm for ARM needs good load balancing.

### 2.3. Partitioning Skew in MapReduce

In a MapReduce application, the outputs of map tasks are distributed among reduce tasks via hash partitioning (by default). In the map phase, the hash partitioning usually takes a hash function *hash*  *key*%*R* to determine the partition number corresponding to each type of key-value pair, where *R* is the number of reduce tasks. The hash function is usually adequate to evenly distribute the data. However, if the outputs are not evenly distributed, hash partitioning may fail with skewed data. This phenomenon is referred to as partitioning skew. For example, in the Inverted Index application, the hash function may partition intermediate data based on the first letter of a word; reducers processing more popular letters are assigned a disproportionate amount of data. Partitioning skew can occur for the following reasons [16]:

1. Skewed tuple sizes: The sizes of values in applications vary significantly, which can lead to uneven workload distribution.
2. Skewed key frequencies: Some keys occur more frequently in intermediate data, causing reduce tasks that process these popular keys to become overloaded.
3. Skewed execution times: Processing a single, large key-value pair may require more time than processing multiple small pairs. Even when the partitioning function perfectly distributes keys across reducers, the execution times of reduce tasks may differ simply because the key groups they are assigned contain significantly more values.

For skewed execution times, we can use domain knowledge when choosing the map output partitioning scheme if the reduce operation is expensive [17]. However, we focus on the other two reasons for significantly longer job execution times that affect the performance of MapReduce. Motivated by the limitations in existing solutions, we use the partition tuning method to disperse key-value pairs in virtual partitions and recombine each virtual partition in case of data skew.

## 3. Partitioning Turning-Based Skew Handling Approach

Based on the virtual partition in the map phase, the repartition in the reduce phase recombines the virtual partitions into new partitions to ensure that the number of reduce tasks is equal to the final number of new partitions. Meanwhile, the size of new data in each partition maintains a certain balance.

### 3.1. Virtual Partitioning in Map Phase

After all map tasks are completed, all key-value pairs are sorted by partition number. Inside the partition, all key-value pairs are sorted following the key order. When dealing with large-scale datasets, the output data generated by each map task usually occupy a large amount of memory, which is spilled to the local disk. All spilled files are then merged and written to the disk after all map tasks are completed. Throughout the process of spilling and merging, the index corresponding to each partition is established by the map tasks. When reading data, it can speed up the task of obtaining subsequent data for the reduce partitions.

In the repartitioning process, the partition results in the map phase are divided and combined once again [17]. The key-value pairs in one partition are hence separated and merged into another. When a reduce task requests partition data based on the results of a new partition, the requested data is distributed in different places in the spilled files, resulting in a nonsequential and inefficient reading of data.

The key challenge in virtual partitioning is choosing the partition number of key-value pairs *R* in function *hash*(*key*)%*R*. By default, *R* is the number of reduce tasks; but, ideally, *R* should be determined by the number of types of input key-value pairs. We think that the appropriate number of virtual partitions is between these two values. When the value of *R* is determined, the partition number is no longer correspondent to the reduce task number through *hash*(*key*)%*R*. The data in each partition in the map phase can be processed by an uncertain reduce task; such a partition is called a virtual partition. Each virtual partition is an integral part of an actual partition that has been repartitioned. The specific relationship is determined by a balancing algorithm once the reduce tasks have all information pertaining to metadata output from the map phase.

The significance of virtual partitions is to disperse the key-value pairs as much as possible, thus providing more combination types for the subsequent repartition process. According to the characteristics of application, system resources, and the degree of dispersion of key-value pairs, the number of virtual partitions *N* can be selected freely by users. To ensure a fair distribution among reducers, we divide the output of all map tasks into virtual partitions. However, the number of virtual partitions may significantly affect the performance of the partitioning phase. When the number of virtual partitions is small, the system can fetch the metadata information of each virtual partition more efficiently. However, fewer virtual partitions can lead to unfair distribution among reducers.

### 3.2. Obtaining Global Output Information

**Figure 2:**
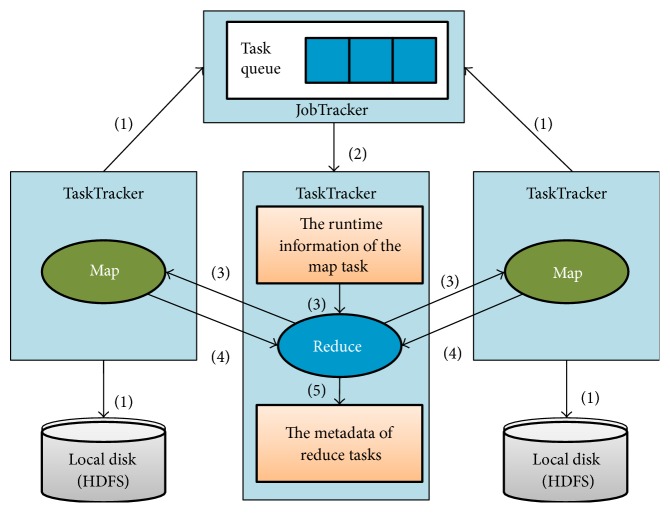
Acquisition of the metadata for reduce tasks.

Based on the global output metadata of map tasks, the repartitioning process makes full use of the communication between map tasks and reduces tasks to divide the original communication process into two phases: (1) obtaining the metadata output of each map task and (2) recombining the information in the reduce tasks.  Figure 2 shows the process of acquisition of metadata for reduce tasks. The detailed steps are as follows:

1. Once all map tasks are complete, the output is written to the local disk. The TaskTracker uses heartbeat information to send messages to a JobTracker stating that the task has been completed.
2. The JobTracker maintains a map task completion message queue for each MapReduce job. When the TaskTracker runs a reduce task asks for a completion message for the map task, the JobTracker removes the message from the queue and delivers it to the corresponding TaskTracker.
3. In the same MapReduce job, a reduce task gets a completion message for the map task from its TaskTracker. The runtime information of the map task is extracted from the completion message, including map task number, and information concerning execution nodes. Using this information, the reduce task establishes an HTTP connection with the execution node and requests the metadata information output of the map task.
4. Based on the request number of a map task, the TaskTracker reads the corresponding index file of the map outputs from the local file system and sends it to the corresponding reduce task.
5. The reduce task merges the virtual partitions of the same index number from different index files. It then aggregates the data of each virtual partition that has the same type of key-value pairs.

### 3.3. Repartitioning

The repartitioning process divides the collected virtual partitions into new partitions of the same number as reduce tasks. The data size of the biggest partition can be minimized after repartitioning process. It can also reduce the processing time needed for the maximum partition, thereby speeding up the completion of the entire reduce phase and increasing the rate of completed jobs as well as system throughput.

As previously analysed, the repartitioning process recombines each virtual partition generated in the map phase. However, due to the limitation of available memory, these virtual partitions must be written to the local file system. If repartitioning is not restricted, it is likely to lead to a plurality of discrete virtual partitions in one partition following the balancing process, resulting in a nonsequential read of the disk. Moreover, in classic algorithm design, the balancing of virtual partitions as a partition problem [18] has been shown to be NP hard and hence impossible to solve in linear time.

In this study, the proposed PTSH adds the following restrictions to the repartitioning process: a new balanced partition must be assembled by the original, continuous, and distributed virtual partitions. The repartitioning result {*P*_1_, *P*_2_, *P*_3_, *P*_4_, *P*_5_} may be {*P*_1_, *P*_2_}, {*P*_3_, *P*_4_}, {*P*_5_}, or {*P*_1_, *P*_2_, *P*_3_}, {*P*_4_, *P*_5_} but cannot be {*P*_1_, *P*_4_}, {*P*_2_, *P*_5_}, {*P*_3_}. Through such constraints, following the repartitioning process, we can ensure that virtual partitions continue to be continuously distributed in spilled files. This also reduces the time complexity of the balancing process. In the repartitioning process, the problem of the recombination of virtual partitions can be described as follows:

(2) $$\begin{array}{c} \begin{array}{c} max\\ 1\;\leq \;i\;\leq n \end{array}a_{i}\;\leq \;S_{max}\leq \overset{n}{\underset{1}{\sum }}{max}_{1\leq i\leq n}^{a_{i}}. \end{array}$$

Define *S*_max_ = mid = (low + high)/2, traverse sequence *A*, and determine the number of subsequences *C*.

When *C* > *K*, *S*_max_ is lower; thus, *S*_max_ should be increased. Hence, define low = mid + 1.

When *C* ≤ *K*, *S*_max_ is higher; thus, *S*_max_ needs to be reduced. Hence, define low = mid, and jump to step 4.

Repeat step 1 till low > mid.

The pseudocode of repartitioning algorithm is as in Algorithm 1.

By applying binary search in the while loop of PTSH, the minimum value of *S*_max_ can be determined. Because it needs to traverse the sequence of integers once in each loop, the time complexity of PTSH is *O*(*N*log∑*A*), where *N* is the number of elements of *A*.

## 4. Evaluation

All experiments to measure the performance of PTSH were performed on a 7-node cluster with six slave nodes and one master node. Each node used two 2 GHz quadcore CPUs with 16 GB of RAM and 500 GB SATA disk drives. All nodes were used as both compute and storage nodes. The HDFS block size was set to 64 MB, and a common gigabit Ethernet switch connected each node. We evaluate PTSH performance on a virtual cluster: five virtual machines were deployed on each of the six machines, reaching a cluster size of 30 data nodes. All virtual machines were configured with one CPU and 1 GB memory. The baseline for our deployment was Hadoop 1.1.2 [19], and we configured the HDFS to maintain three replicas for each data block in this cluster.

### 4.1. Measures of Data Skewness and Data Locality

Some distributions of data, such as the bell curve, are symmetric. This means that the right and the left parts of the distribution are perfect mirror images of each other. Not every distribution of data is symmetric. We know that data skew arises out of the physical properties of objects and hotspots on subsets of the entire domain (e.g., the word frequency appearing in documents obeys a Zipfian distribution). The measure of how asymmetric a distribution is is called skewness and is used as a fairness metric in the literature [20]. We use the coefficient of variation to numerically calculate the measure of data skewness as follows:

(3) $$\begin{array}{c} Cov=\frac{stdev}{mean}\times 100\%. \end{array}$$

The data distribution is completely fair if the coefficient of variation is zero. As Cov increases, skewness does as well.

Data locality is important for performance evaluation. In this paper, data locality is the sum of the frequencies of keys in nodes, which are partitioned to that of the frequencies of all keys [5]:

(4) $$\begin{array}{c} \begin{array}{c} {Locality}_{min}=\frac{\sum_{i=1}^{K}{min}_{1\leq j\leq n}{FK}_{i}^{j}}{\sum_{i=1}^{K}{FK}_{i}},\\ {Locality}_{max}=\frac{\sum_{i=1}^{K}{max}_{1\leq j\leq n}{FK}_{i}^{j}}{\sum_{i=1}^{M}{FK}_{i}}, \end{array} \end{array}$$

where min_*i*≤*j*≤*n*_*FK*_*i*_^*j*^ indicates the minimum frequency of key *k*_*i*_ in data node *n*^*j*^ and max_*i*≤*j*≤*n*_*FK*_*i*_^*j*^ is the maximum frequency of key *k*_*i*_ in data node *n*^*j*^.

### 4.2. Performance of PTSH on Applications

First, to compare native Hadoop and PTSH, we performed our evaluations on PUMA [21], which represents a wide range of MapReduce applications exhibiting characteristics with high/low computation and high/low shuffle volumes. Second, we evaluated PTSH with Closer [9], LEEN [5], and native Hadoop through the Word Count application. The applications used in our evaluation were as follows:

1. Inverted Index (II): It takes a list of documents as inputs and generates word-to-document indexing. Map emits <word, docId> tuples with each word emitted once per docId. Reduce combines all tuples on key <word> and emits <word, list(docId)> tuples after removing duplicates.
2. Word Count (WC): This application counts the occurrences of each word in a large collection of documents. Map emits <word,1> tuples. Reduce adds the counts for a given word from all map tasks and outputs the final count.

Table 1 gives an overview of these applications together with the configurations we used in our experiments. We used Wikipedia data [22] for Inverted Index and generated skewed data by RandomWriter [23] for Word Count. In our experiments, we used the frequency variation of the keys and their distribution as parameters in the motivation of the design. Since the former clearly causes variation in the data distribution of the inputs of the reducers, the variation in the latter affects the amount of data transferred during the shuffle phase [5]. We present the results of executing these applications with varying sizes of input data, frequency of variation of the keys, and average variation in key distribution. We ran each application at least five times and used the average performance results.

The number of virtual partitions depends on the tuning ratio (TR) set by user, which can be computed as follows:

(5) $$\begin{array}{c} TR=\frac{V}{R}. \end{array}$$

In the above, *V* is the number of virtual partitions and *R* is the number of reduce tasks. To compare the proposed algorithm with the native Hadoop system, we ran each application by using the PTSH algorithm with different partition turning parameters. The value of TR varied from 1 to 50. When TR = 1, this meant that PTSH was not used and reached the uniform distribution of each key among the data nodes (key  distribution  variation = 0%). However, in the map phase, the combining process affected the amount of data to be transferred during the shuffle phase, emphasising the amount of input data for the reduce tasks. Therefore, the map native combine was not a factor in our experiments.

**Figure 3:**
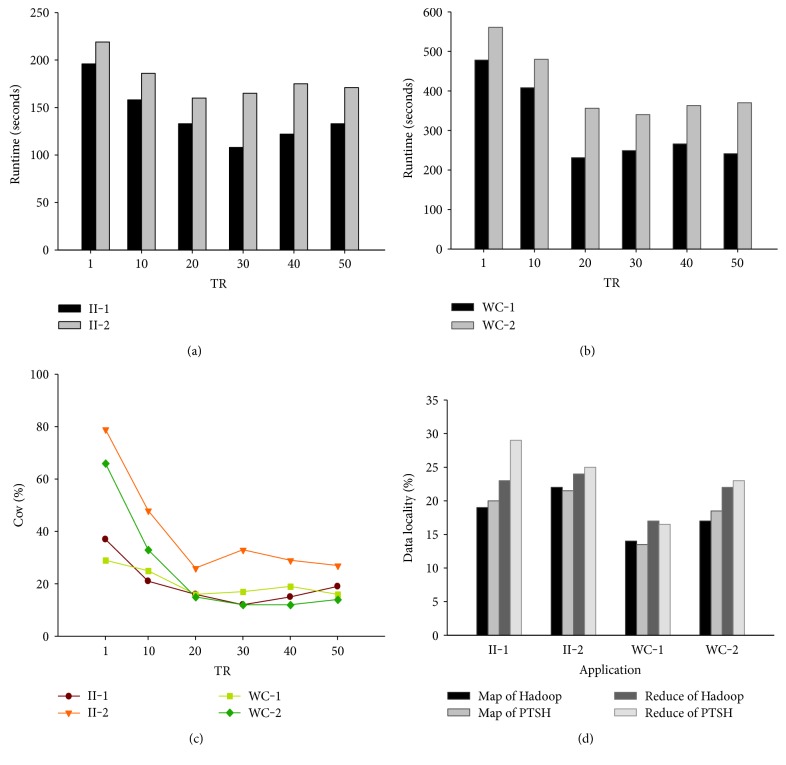
Performance of II and WC with different variations in the frequency of keys as well as different key distributions.

Figure 3 shows the performance of II and WC. As shown in Figure 3(a), for II-2, in the best case (TR = 20), the runtime was 1.24x faster than the native Hadoop system. II-1 achieved better performance improvement when TR was 30, when it increased to 1.61x. For both II-1 and II-2, even in the worst case, the runtime decreased by 12.7% (TR = 10) and 11.5% (TR = 10), respectively.  Figure 3(b) shows the performance of WC. WC-1 ran 1.12–1.57x faster than native Hadoop, and the promotion of WC-2 varied in the range from 13.1% to 25.4%.  Figure 3(c) shows that the coefficient of variation could be effectively controlled by PTSH by using virtual partitioning. The map locality of PTSH did not achieve better performance than that of native Hadoop.  Figure 3(d) also shows that the promotion of the map locality of PTSH varied in the range from −2.3% to 5.4%, and that of the reduce locality varied from −1.7% to 13.5% due to the recombination of the virtual partitions. We think that the virtual partitioning in the map phase separated the tuples into many virtual partitions, which affected the performance of map locality. We think that data locality varied considerably (different runs of the same workload might have resulted in different data localities). The PTSH is designed to achieve a better-balanced distribution of the reducers' inputs than data locality. In Table 2, we see that PTSH achieved better fairness in the reducers' inputs between nodes than native Hadoop, which in turn resulted in balanced reduce function executions. All reducers therefore finished nearly at the same time. This experiment showed that with PTSH, the runtime of each application decreased clearly, and data locality was stable when data skew occurred. Of each pair of applications, in cases involving large frequencies of variation in keys and a higher distribution, the performance of the proposed application was better than that in other cases. However, as the figures show, an increase in TR added overhead to the system and performance was stable when TR was in the range 10 to 40. We then used Word Count to evaluate the performance of PTSH (TR = 30) against data skew compared with native Hadoop, Closer, and LEEN. Since the operation of Word Count on reducer was only the addition operation, we selected fixed reducers (reducer = 60) to compare performance and chose (1) the detailed runtime of each stage, (2) the runtime of the worst and best tasks, (3) the coefficient of variation, and (4) data locality range in the shuffle phase as indicators.

**Figure 4:**
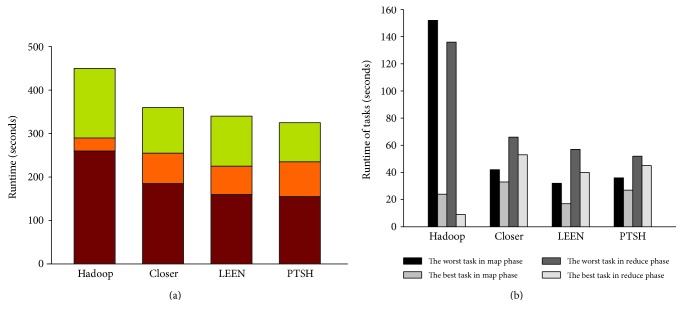
Performance of WC-3 with native Hadoop, Closer, LEEN, and PTSH.

Regarding the runtime of the entire job, PTSH outperformed native Hadoop, Closer, and LEEN in WC-3. As shown in Figure 4(a), PTSH outperformed native Hadoop, Closer, and LEEN by 27.5%, 7.3%, and 4.9%, respectively: the runtime of shuffle phase in PTSH was longer than that in other methods due to the repartitioning algorithm, but the runtime of the reduce phase in PTSH achieved the best performance (the time taken by the best reduce function was 92 s and that by the worst reduce function was 160 s), due to the better fairness in the data distribution of the reducers' inputs (as shown in Table 3, PTSH achieved better Cov than other methods).  Figure 4(b) shows that when using PTSH, the runtime of the best task and the worst task in the map and reduce phases achieved better performance than those in native Hadoop, Closer, and LEEN (the time taken by the best task and the worst task in the map phase was 37 s and 26 s, and the time taken by the best task and the worst task in the reduce phase was 52 s and 43 s). Thus, PTSH effectively handled the skew of reducers by mitigating the unbalance of tasks.

### 4.3. Case Study

At present, data generated by health organizations is vast and complex and makes it difficult to analyse to make important decisions regarding patient health. This data contains details regarding hospitals, patients, medical claims, treatment cost, and so forth Thus, there is a need to generate a powerful tool to analyse and extract important information from this complex data. The analysis of healthcare data improves healthcare by enhancing the performance of patient management tasks. The outcome of data mining technologies is to provide benefits to healthcare organization in grouping patients with similar diseases or health issues to provide them with effective treatment.

To improve the performance of association mining on healthcare data with MapReduce, we used the Apriori [24] algorithm to analyse healthcare data from the National Survey on Drug Use and Health (NSDUH) [25], 2004–2014, which primarily measured the prevalence and correlation of drug use in the United States. The surveys were designed to provide quarterly as well as annual estimates. Information was provided on the use of illicit drugs, alcohol, and tobacco among US residents aged 12 and older. Questions included age at first use; lifetime, annual, and past-month usage for the following drug classes: cannabis, cocaine, hallucinogens, heroin, inhalants, alcohol, and tobacco; nonmedical use of prescription drugs including psychotherapeutics; and polysubstance use. Respondents were also asked about their knowledge of drugs, perceptions of the risks involved, population movement, and sequencing of drug use. We conducted data preprocessing on this dataset and removed irrelevant information. The detailed characteristics of the dataset were as in Table 4.

As the classic ARM algorithm, the Apriori algorithm can obtain knowledge with important reference value for decisions and judgments and, hence, can be used to mine healthcare data. However, the traditional Apriori algorithm has three shortcomings: (1) It can mine incorrect strong association rules. (2) The cost of the algorithm fluctuates greatly when only using the degree of support as the determinant of candidate set generation. (3) When dealing with a large amount of data, the system I/O load increases and processing speed slows down, seriously affecting the algorithm's efficiency. In this case study, we used the interest measure-based Apriori algorithm (IM-Apriori) [26] to efficiently mine strong association rules. Algorithm 2 summarizes the IM-Apriori algorithm on MapReduce. We also combined PTSH and IM-Apriori to improve the performance of MapReduce in case of data skew in NSDUH.

**Figure 5:**
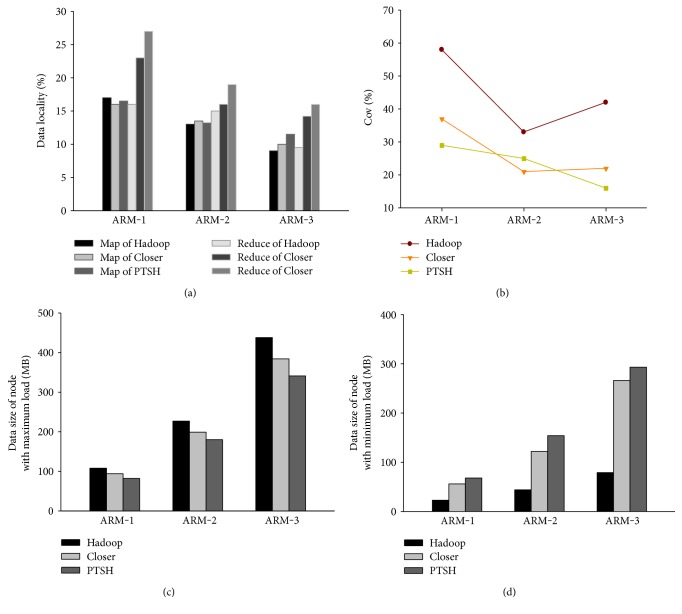
Performance of data locality, Cov, and the data size of nodes with maximum and minimum load for the three tests.

**Figure 6:**
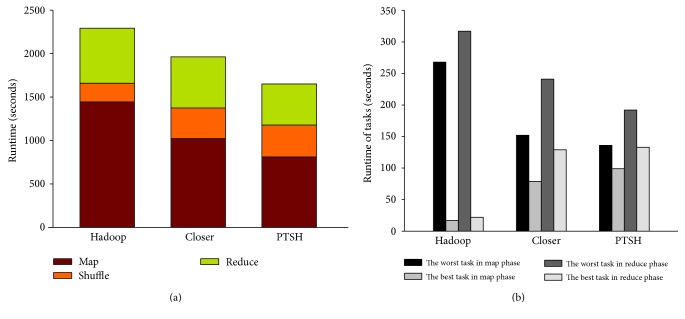
Detailed performance of each stage and tasks in Hadoop, Closer, and PTSH for ARM-3.

We evaluated the IM-Apriori algorithm on subsets of NSDUH with native Hadoop, Closer, and PTSH. For IM-Apriori, the parameters of the association rule can be seen in Table 5. This study used an 11-node real cluster and the same hardware configuration as before. Since the transaction size was large, we needed to divide them into blocks and distribute to different data nodes. The number of reducers was set to 20, and the TR we used in PTSH was 30.  Table 6 shows the performance of IM-Apriori algorithm when using native Hadoop, Closer, and PTSH. When the size of the transaction increased, the differences among the three methods were greater. In the best case, PTSH ran 1.44x and 1.20x faster than native Hadoop and Closer, respectively.  Figure 5 shows the performance of data locality, Cov, and the data size of nodes with maximum and minimum load for the three tests. As we can see in Figure 5(a), the reduce locality of PTSH also outperformed its map locality and the map localities of these tests achieved almost the same locality. This can be explained due to the better Cov in Figure 5(b) and fairness in data distribution of reducers' inputs in Figures 5(c) and 5(d).  Figure 6 showed the detailed performance of each stage and tasks in Hadoop, Closer, and PTSH for ARM-3. As shown in Figure 6(a), the latency of the map phase in native Hadoop was higher than that of Closer and PTSH due to the map skew and the latency of the shuffle phase in PTSH was longer than that in other methods due to the repartitioning algorithm. However, as shown in Figure 6(b), the better fairness in reducers' inputs data between nodes in PTSH resulted in balanced reduce task executions, which in turn made all reducers finished almost at the same time. The IM-Apriori algorithm is hence more suitable for healthcare data mining than native Hadoop and Closer.

In Table 7, we list some of the results of association rule mining from NSDUH. We found that an average young adult smoker tended to drink alcohol; the confidence of this rule was 0.72. On the other hand, we also found that divorced women also belonged to the group of smokers, as did many unemployed adults. With the rapid changes in society, social pressure is increasing and more and more people smoke and drink, which has a significant impact on human health. Through the analysis of data association rules, we can find some common characteristics. According to these characteristics, we can offer the relevant guidance and help to prevent people from excessive drinking and smoking.

## 5. Conclusions

Big data is changing how we live in many ways, such as shopping, relationships, and education. One of the most promising areas where big data can be applied for improvements is healthcare. The fields of medical and health generate large volumes of data, for instance, electronic medical records. Both the volume and the velocity of data in healthcare are truly sufficiently high to require big data today. Understanding these data with methodologies using big data processing can help analytics for clinical improvements, financial analysis, and fraud and waste monitoring.

This paper proposed a PTSH algorithm to balance the input data of reduce tasks, which aims to process data in healthcare-related areas. Performance studies carried out on a seven-node MapReduce cluster showed that PTSH outperformed native Hadoop, Closer, and LEEN. Compared with one-stage partitioning strategies, two-stage partitioning can mitigate skew data in reduce tasks. It was found that data skewness and workload balance simultaneously influenced the efficiency of MapReduce. Our analysis and experimental results showed that MapReduce is sensitive to workload balance, although good skewness is also important. MapReduce was effective in the best case of high balance and high skewness. The combination of high balance and moderate skewness was the second-best case.

Large data analysis provides a new approach to the resolution of many healthcare problems, where mining small data cannot help extract valuable information to serve economic and social development. As healthcare data continues to increase in size, there is a need to generate a powerful tool to analyse and extract important information from these complex data. A case study of ARM for NSDUH data was carried out on MapReduce. The results showed that workload balance is important for ARM on MapReduce, as it ensures the minimum execution time for the reduce phase. We discovered that two-stage partitioning performed better, and PTSH improved the efficiency of ARM on real healthcare data. We think that even if healthcare data is seriously skewed, good workload balance can better solve the problem.

The current strategy requires obtaining all metadata outputs by map tasks before the reduce phase. However, in processing applications of large-scale data, overhead due to transmission between the map phase and the reduce phase may increase. In future work, we plan to focus on optimizing this overhead to achieve better balance performance.

## Conflicts of Interest

The authors declare that they have no competing financial interest.

## Figures and Tables

**Algorithm 1:**
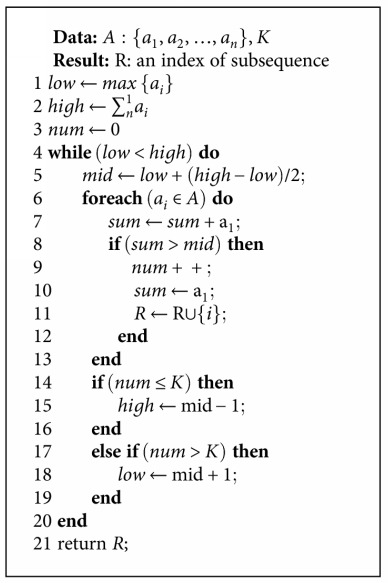
Repartitioning algorithm.

**Table 1:** Application characteristics.

Application	Data type	Input data size (GB)	Frequency of variation of the keys	Average variation in key distribution	Method
II-1	Wikipedia	4	61%	33%	Hadoop, PTSH
II-2	Wikipedia	4	156%	108%	Hadoop, PTSH
WC-1	RandomWriter	7.5	42%	136%	Hadoop, PTSH
WC-2	RandomWriter	7.5	125%	211%	Hadoop, PTSH
WC-3	RandomWriter	7.5	116%	130%	Hadoop, Closer, LEEN, PTSH

**Table 2:** The detailed performance of the nodes with maximum and minimum load.

Application	Method	Node with maximum load		Node with minimum load	
		Size (MB)	Runtime (seconds)	Size (MB)	Runtime (seconds)
II-1	Hadoop	292	75	27	8
	PTSH	187	49	122	33

II-2	Hadoop	329	92	15	5
	PTSH	205	66	107	36

WC-1	Hadoop	391	154	43	21
	PTSH	278	106	230	82

WC-2	Hadoop	425	180	12	3
	PTSH	291	119	195	71

**Table 3:** Data locality and Cov of each method.

Method	Cov	Locality range
Hadoop	79%	3%
Closer	23%	1–12%
LEEN	15%	1–16%
PTSH	11%	1–14%

**Table 4:** Dataset characteristics.

Attribute characteristic	Transaction size (GB)	Numbers of instances	Numbers of attributes
Categorical, integer	3.31	4,905,142	247

**Table 5:** Association rule parameters.

Parameter name	Parameter value
Minimum confidence	0.6
Minimum support	0.2
Minimum interest	0.3

**Table 6:** Performance of IM-Apriori algorithm.

	Transaction size (GB)	Number of blocks	Runtime of native Hadoop (seconds)	Runtime of Closer (seconds)	Runtime of PTSH (seconds)
ARM-1	0.72	10	914	795	706
ARM-2	1.68	20	1522	1147	1058
ARM-3	3.31	40	2359	1962	1632

**Table 7:** Association rules for NSDUH (2004–2014).

No.	Rules	Confidence
1	Age = young adult and smoking = more ≥ alcohol = yes	0.72
2	Gender = male and marital status = divorce ≥ cigarette = yes	0.65
3	Job status = unemployment and age = older adult ≥ alcohol = yes	0.63

## References

[1] Raghupathi W., Raghupathi V. (2014). Big data analytics in healthcare: promise and potential. *Health Information Science and Systems*.

[2] Dikaiakos M. D., Katsaros D., Mehra P., Pallis G., Vakali A. (2009). Cloud computing: distributed internet computing for IT and scientific research. *IEEE Internet Computing*.

[3] Shim K. (2012). MapReduce algorithms for big data analysis. *Proceedings of the VLDB Endowment*.

[4] Kwon Y. C., Balazinska M., Howe B., Rolia J. A study of skew in mapreduce applications.

[5] Ibrahim S., Jin H., Lu L., He B., Antoniu G., Wu S. (2013). Handling partitioning skew in mapreduce using leen. *Peer-to-Peer Networking and Applications*.

[6] Xu Y., Zou P., Qu W., Li Z., Li K., Cui X. Sampling-based partitioning in MapReduce for skewed data.

[7] Ramakrishnan S. R., Swart G., Urmanov A. Balancing reducer skew in MapReduce workloads using progressive sampling.

[8] Lin J. (2009). The curse of zipf and limits to parallelization: a look at the stragglers problem in mapreduce. *7th Workshop on Large-Scale Distributed Systems for Information Retrieval*.

[9] Gufler B., Augsten N., Reiser A., Kemper A. Handing data skew in MapReduce.

[10] White T. (2012). *Hadoop: The definitive guide*.

[11] Tomar D., Agarwal S. (2013). A survey on data mining approaches for healthcare. *International Journal of Bio-Science and Bio-Technology*.

[12] Ji Y., Ying H., Tran J. Mining infrequent causal associations in electronic healthcare databases.

[13] Patil B. M., Joshi R. C., Toshniwal D. Association rule for classification of type-2 diabetic patients.

[14] Abdullah U., Ahmad J., Ahmed A. (2008). Analysis of effectiveness of apriori algorithm in medical billing data mining. *ICET*.

[15] Zaki M. J. (1999). Parallel and distributed association mining: a survey. *IEEE Concurrency*.

[16] Liu Z., Zhang Q., Boutaba R., Liu Y., Wang B. (2016). OPTIMA: on-line partitioning skew mitigation for MapReduce with resource adjustment. *Journal of Network and Systems Management*.

[17] Yang H. C., Dasdan A., Hsiao R. L., Parker D. S. Map-reduce-merge: simplified relational data processing on large clusters.

[18] Chopra S., Rao M. R. (1993). The partition problem. *Mathematical Programming*.

[19] Hadoop [EB/OL]. http://lucene.apache.org/hadoop.

[20] Jain R., Chiu D. M., Hawe W. R. (1984). *A Quantitative Measure of Fairness and Discrimination for Resource Allocation in Shared Computer System*.

[21] Ahmad F., Lee S., Thottethodi M., Vijaykumar T. N. (2012). *Puma: Purdue Mapreduce Benchmarks Suite*.

[22] Urdaneta G., Pierre G., Steen M. V. (2009). Wikipedia workload analysis for decentralized hosting. *Computer Networks*.

[23] Hammoud M., Rehman M. S., Sakr M. F. Center-of-gravity reduce task scheduling to lower mapreduce network traffic.

[24] RAgrawal R., Srikant R. Fast algorithms for mining association rules.

[25] United States Department of Health and Human Services. Substance Abuse and Mental Health Services Administration. Center for Behavioral Health Statistics and Quality (2016). *National Survey on Drug Use and Health, 2014, ICPSR36361-v1*.

[26] Omiecinski E. R. (2003). Alternative interest measures for mining associations in databases. *IEEE Transactions on Knowledge and Data Engineering*.

